# Ileal Digestibility and Total Tract Retention of Phosphorus in Inorganic Phosphates Fed to Broiler Chickens Using the Direct Method

**DOI:** 10.3390/ani10112167

**Published:** 2020-11-20

**Authors:** Su Hyun An, Jung Yeol Sung, Changsu Kong

**Affiliations:** 1 Department of Animal Science and Biotechnology, Kyungpook National University, Sangju 37224, Korea; woobi89@gmail.com; 2 Department of Animal Sciences, Purdue University, West Lafayette, IN 47907, USA; sung73@purdue.edu; 3 Department of Animal Science, Kyungpook National University, Sangju 37224, Korea

**Keywords:** phosphorus, direct method, ileal digestibility, inorganic phosphates, total tract retention, broilers

## Abstract

**Simple Summary:**

There is limited information on the phosphorus (P) utilization of inorganic phosphates determined using the direct method in broiler chickens. The present study aimed to determine the ileal digestibility and total tract retention of P in several inorganic phosphates in broiler chickens using the direct method. No interaction was observed between the P source and the collection site (ileal digestibility vs. total tract retention). Phosphorus utilization differed among inorganic phosphates, and the ileal digestibility of P was greater than the total tract retention. Based on the current findings, different P utilization values should be applied to each inorganic phosphate.

**Abstract:**

The objective of this study was to determine and compare the phosphorus (P) utilization of inorganic phosphates fed to broiler chickens using the direct method. On day 15 of age, six hundred forty 15-day-old male Ross 308 broiler chickens (initial body weight = 399 ± 38 g) were assigned to five experimental diets with 16 birds per cage in a randomized complete block design. The experimental diets consisted of four semi-purified diets containing monocalcium phosphate, monodicalcium phosphate, dicalcium phosphate, and tricalcium phosphate as the sole P sources. Additionally, a P-free diet was prepared to measure basal endogenous P loss. Chromic oxide was added to the experimental diets as an indigestible index. Excreta were collected per cage on days 17 to 18 of age, and all birds were asphyxiated with carbon dioxide on day 19 of age for ileal digesta collection. The cage was an experimental unit, and the number of replications per each treatment was eight except for the tricalcium phosphate treatment (n = 4). There was no interaction observed between the P source and the collection site (ileal digestibility vs. total tract retention). Phosphorus utilization differed (*p* < 0.05) among inorganic phosphates and the ileal digestibility of P was greater (*p* < 0.05) than the total tract retention. In conclusion, the standardized ileal digestibility of P in inorganic phosphates ranged from 56.7% to 89.8% and ileal digestibility was greater than the total tract retention.

## 1. Introduction

Phosphorus (P) is an essential nutrient for nearly all forms of life. In poultry, P is involved in physiological and metabolic pathways including skeleton mineralization, energy metabolism, and nucleic acid synthesis [1]. Most P in plant-derived feed ingredients used in poultry and swine diets is organic P which is bound to phytate. Poultry possess effective phytase activity that can liberate inorganic P from phytate, but they cannot efficiently utilize P in plant-based feedstuffs due to the poor solubility of phytate in the gastrointestinal tract [2]. For this reason, inorganic phosphates are widely used in poultry diets to meet the P requirements of animals [3].

The bioavailability of inorganic phosphates can be determined by the slope-ratio method [4,5]. However, the slope-ratio method frequently results in a large standard error because the relative bioavailability values of the reference sources may differ. Furthermore, a value obtained by the slope-ratio method is a comparative value that challenges nutritionists to formulate diets. For these reasons, ileal (pre-cecal) digestibility and retention have been suggested to accurately represent the bioavailability of P in inorganic phosphates in poultry diet formulation [6].

The ileal digestibility and retention of P in inorganic phosphates should be accurately determined because the application of inaccurate utilization values in mixed diets may lead to economic loss and environmental pollution [7]. Linear regression analysis is regarded as a standard method for poultry to determine the ileal digestibility and retention of P in inorganic phosphates, with an assumption that no interaction between the basal diet and relative P sources exists [6,8,9,10]. However, the regression method requires various diets and compositions in basal diets that may affect P availability in the test ingredients. In contrast to the regression method, the direct method only requires a P-free diet and a diet where a test ingredient is the sole source of a component of interest [11]. Furthermore, ileal digestibility and retention determination using the direct method are less likely to be affected by basal diet types because the direct method uses semi-purified diets. To our knowledge, there is limited information on the determination of the ileal digestibility and retention of P in inorganic phosphates using the direct method. Therefore, the objective of the present study was to determine the ileal digestibility and total tract retention of P in several inorganic phosphates fed to broiler chickens using the direct method.

## 2. Materials and Methods

All protocols used in the study were approved by the Animal Care and Use Committee of Kyungpook National University (Approval number: KNU 2019-125).

### 2.1. Birds, Diet and Experimental Design

A total of 640 day-old Ross 308 male broiler chickens were used in the current assay. Birds were housed in battery cages (60 × 60 × 60 cm) and fed a starter and grower diet until 7 and 15 d of age, respectively. At day 15 of age, all birds [initial body weight (BW) = 399 ± 38 g] were individually tagged, weighed, and assigned to 5 experimental diets in a randomized complete block design, with BW as a blocking factor using a spreadsheet program [12]. The experimental diets consisted of 4 semi-purified diets containing monocalcium phosphate [MCP; Ca(H_2_PO_4_)_2_], monodicalcium phosphate [MDCP; Ca(H_2_PO_4_)_2_ + CaHPO_4_], dicalcium phosphate (DCP; CaHPO_4_), and tricalcium phosphate [TCP; Ca_3_(PO_4_)_2_] as the sole source of P in each diet (Table 1 and Table 2). A P-free diet was additionally prepared to measure the basal endogenous loss (BEL) of P. The particle size of the inorganic phosphates and limestone that were used in the present study was between 500 and 1000 µm. All diets were formulated to meet the requirements of amino acids [13], Ca, and P [14], except for the P-free diet where P was deficient. All experimental diets contained 0.5% chromic oxide as an indigestible index. Each dietary treatment consisted of 8 cages with 16 chickens.

### 2.2. Animal Management

All birds had *ad libitum* access to water and feed from days 0 to 19 of age. Birds were housed in wire-floored cages in an environmentally controlled room at 33 °C for the first 3 days, and then temperature was gradually decreased by 3 °C per week to 24 °C by day 19 of age according to genetic strain recommendations.

### 2.3. Sample Collection

Birds had free access to the experimental diets and water from days 15 to 19 of age. Excreta were totally collected from days 17 to 18 of age. On day 19 of age, individual BW and feed leftovers were recorded, and birds were asphyxiated with carbon dioxide for ileal digesta collection. Ileal digesta were collected from the distal two-thirds section of the ileum (a portion of the small intestine from Meckel’s diverticulum to approximately 1 cm anterior to the ileo–cecal junction) by flushing with distilled water. The pooled excreta and ileal digesta samples per cage were stored at −20 °C and dried in a forced-air oven (JSOF-150, JS research, Gongju, Korea) at 55 °C.

### 2.4. Chemical Analyses

Dry matter (DM) [15] and chromium [16] concentrations in diets were determined. The gross energy of the diets was determined using a bomb calorimeter (Parr 6400, Parr Instruments Co., Moline, IL, USA). The diets and inorganic phosphates were analyzed for Ca (method 935.13) and P concentrations (method 946.06) [17]. Dried excreta and ileal digesta samples were also analyzed for P and chromium concentrations.

### 2.5. Calculations and Statistical Analyses

Apparent ileal digestibility (AID, %), apparent total tract retention (ATTR, %), BEL [g/kg of DM intake (DMI)] of P at the ileum and total tract, standardized ileal digestibility (SID, %), and standardized total tract retention (STTR, %) were calculated using the following equations [18]:

AID or ATTR (%) = [1 − (Cr_i_ ÷ Cr_o_) × (P_o_ ÷ P_i_)] × 100

BEL of P at ileum and total tract (g/kg of DM intake) = (Cr_i_ ÷ Cr_o_) × P_o_

SID or STTR (%) = AID or ATTR + (BEL of P ÷ P_i_) × 100

where Cr_i_ and Cr_o_ represent the chromium concentration (g/kg DM) in the diet and ileal digesta and excreta, respectively, and P_i_ and P_o_ represent P concentration (g/kg DM) in the diet and ileal digesta and excreta, respectively.

Data were analyzed using the MIXED procedure of the SAS software (SAS Inst. Inc., Cary, NC, USA). Dietary treatments were considered as a fixed variable, whereas the block was considered as a random variable. The least squares means were calculated for each treatment. For growth performance, differences between least squares means were tested using the PDIFF option with Tukey′s adjustment. Orthogonal contrasts were used to determine the main effect of the P source and collection site (ileal digestibility or total tract retention), as well as the interaction between the two main effects [19]. The statistical model of the current study is as follows:

Y_ij_ = μ + Τ_i_ + C_j_ + ΤC_ij_ + ε_ij_

where Y_ij_ is the response, μ is the overall mean, Τ_i_ is the effect of P sources, C_j_ is the effect of collection site, ΤC_ij_ is the interaction between P sources and collection site, and ε_ij_ is the error. As the interaction was not observed in the response criteria, the results were interpreted using only the main effects, and differences between least squares means for P utilization in inorganic P sources were also tested using the PDIFF option with Tukey′s adjustment. The BEL of P at the ileum and total tract were compared using a paired *t*-test. The experimental unit was the cage, and an alpha level of 0.05 was used to determine statistical significance. For the TCP diet, four observations were excluded in the statistical analysis because the ileal digestibility and total tract retention values of these observations were negative or nearly zero, which was not physiologically reasonable.

## 3. Results

The final BW and average daily gain in broiler chickens fed the MCP diet were greater (*p* < 0.05) than those of chickens fed the DCP and TCP diets (Table 3). The average daily feed intake in chickens fed the MCP diet was greater (*p* < 0.05) than that in chickens fed the DCP diet. The ileal digestibility and total tract retention of P in inorganic phosphates are shown in Table 4 and Table 5. An interaction between the inorganic P source and collection site was not observed. The AID, ATTR, SID, and STTR of P in the TCP group were less (*p* < 0.05) than those in the MCP, MDCP, and DCP groups. The ileal digestibility of P in inorganic phosphates was greater (*p* < 0.05) than the total tract retention. The BEL of P at the ileum was less (*p* < 0.05) than that at the total tract. Both the apparent and standardized utilization of P in MCP, regardless of the collection site, were greater (*p* < 0.05) than those in the DCP and TCP groups.

## 4. Discussion

Both ileal digestibility and retention are common methods for estimating P bioavailability in poultry [6]. However, the ileal digestibility of P may be advantageous over total tract retention in poultry because the linear response in ileal P digestibility to dietary P is observed in a wider range when a comparison is made with the total tract retention method, which may be biased because urinary P excretion is not considered when determining ileal digestibility [6].

The regression method is regarded as a standard method for determining the ileal digestibility and retention of P in poultry [8]. The direct method has several advantages over the regression method although the semi-purified diets that are used in the direct method are less practical. The direct method only requires a P-free diet and a diet where a test P source is the sole source [11]. In contrast, the regression method suggested by World’s Poultry Science Association [8] requires a basal diet and diets supplemented with at least two concentrations of a test P source, making it more expensive and laborious. Furthermore, the negative endogenous loss of P can be estimated using the regression method due to its inherent limitation, which is not physiologically possible in chickens [20,21].

Additionally, the ileal digestibility and retention of P in inorganic phosphates determined using the direct method are less likely to be affected by diet-related factors because the semi-purified diets that are used in the direct method are based on cornstarch, sucrose, and gelatin that contain small amounts of intrinsic phytase, phytates, and non-starch polysaccharides. However, in the regression method, P availability may be affected by basal diet components due to the differences in the aforementioned factors [10]. In the study of Shastak et al. [10], ileal P digestibility of MCP in corn- and wheat-based diets fed to broiler chickens, as determined using the regression method, were not different (53% vs. 55%). However, as the dietary anti-nutrient concentrations in the study of Shastak et al. [10] were not sufficient to affect P digestibility, P utilization can be potentially influenced by basal diet types. From this point of view, the P digestibility of inorganic phosphates has been determined using the direct method in pigs [22,23]. However, information on the ileal digestibility of P in several inorganic phosphates fed to broiler chickens determined using the direct method is very limited.

Calcium and P concentrations in inorganic phosphates used in the present study, except for TCP, were within the range of reported values [9,10,23,24]. The calcium concentration of TCP used in the present study (38.4%) was greater than reported values (30.7 to 34.6%) [23,25,26], whereas P concentration (20.1%) was comparable with that reported in previous studies (18.0 to 20.1%).

In the current study, the average daily gain of chickens was nearly zero or negative, which may have been due to a low feed intake resulting in an insufficient supply of essential growth nutrients. The low feed intake may have been partially due to the semi-purified diets used in the study. A greater feed intake of the MCP diet compared with the DCP diet was observed in the present work. The current result agrees with that of Bikker et al. [25], but no difference was observed in other studies [3,23,27]. The discrepancy in feed intake is difficult to explain because the factors affecting feed intake in previous studies were similar to those in the present study.

The ileal digestibility and retention of P in various inorganic phosphates for broiler chickens have been reported in previous studies [3,9,10,25,27,28]. A high dietary Ca to non-phytate P ratio is known to negatively affect P utilization due to the formation of insoluble Ca–P complexes in the intestinal lumen, which are not biologically available for chickens [29,30]. For this reason, the dietary Ca to non-phytate P ratio of the experimental diets in the present study, except for the P-free diet, was fixed at 2.2, as suggested by National Research Council [14], to avoid the potential effects of the dietary Ca to non-phytate P ratio on P utilization. The numerical ranking of P utilization among the four inorganic phosphates in the current study (MCP > MDCP > DCP > TCP) agrees with that in broiler chickens [25] and pigs [23]. As MDCP is a mixture of MCP and DCP, it is logical that the P utilization of MDCP was intermediate between MCP and DCP. However, the direct comparison between the P utilization determined in the present study and reported values is difficult due to different ratios of anhydrous and dihydrous P forms in inorganic phosphates, experimental methodology (direct, regression, and difference method), age of broilers, feeding period, and particle size.

In the present study, the ileal digestibility of P in inorganic phosphates was greater than the total tract retention of P. The differences between the ileal digestibility and total tract retention of P were attributed to post-ileal P absorption, post-ileal P excretion, urinary P excretion, or all of these [9]. As P absorption and secretion posterior to the ileum rarely occur in chickens [31], the discrepancy between ileal digestibility and retention may have been from urinary P excretion. Urinary P excretion is influenced by non-phytate P intake, Ca intake, and the dietary Ca to non-phytate P ratio [6]. In the study of Gautier et al. [29], the bone breaking force and tibia ash of broiler chickens fed different amounts of Ca (0.4 to 1.6% of diet) and non-phytate P (0.2 to 0.8% of diet) with the fixed dietary Ca to non-phytate P ratio differed. In the current study, the dietary Ca to non-phytate P ratio was constant among chickens, and the average daily feed intake of chickens (18.0 to 20.4 g/d) was very low, resulting in a low intake of Ca and P. For this reason, the mobilization of Ca and P from bone was expected to occur in the broilers to maintain homeostasis [32,33]. A portion of the mobilized P was excreted via excreta, which may have led to a greater ileal digestibility compared with total tract retention in the current study.

The BEL of P at the ileum (145 mg/kg DMI) and at the total tract (433 mg/kg DMI) in the present study were within the range of reported values (25 to 446 mg/kg DMI and 51 to 830 mg/kg DMI, respectively) [20,34,35,36,37,38]. The wide range of the BEL of P in previous studies was partially attributed to the different ingredients in the diets. In the study of Ravindran and Mutucumarana [37], the BEL of P at the ileum in broilers fed with three types of P-free diets (protein- and P-free diet, gelatin-based diet, and casein-based diet) was 25, 104, and 438 mg/kg DMI, respectively. Enzyme secretions in broilers fed with the protein- and P-free diets may have been reduced, possibly resulting in decreased endogenous P secretion [37]. Furthermore, as the biological value of casein is greater than that of gelatin in chickens [39], it was speculated that protein retention may be greater in broilers fed with the casein-based P-free diet, resulting in greater proteolytic enzyme secretion and, subsequently, a higher BEL of P compared with chickens fed with the gelatin-based P-free diet [37,40]. The BEL of P at the ileum in the current study and the value derived from the gelatin-based diet reported by Ravindran and Mutucumarana [37] were comparable (145 and 104 mg/kg DMI, respectively) because of the similarity in diet composition.

In the current study, the BEL of P at the total tract was greater than that at the ileum (433 vs. 145 mg/kg DMI, respectively), which agrees with the results of the study by Ravindran and Mutucumarana [37] (560 vs. 104 mg/kg DMI, respectively). As a part of the mobilized P from the bones in chickens fed with the P-free diet was excreted via excreta, the BEL of P at the total tract was expected to be greater than that at the ileum. The lower BEL of P at the total tract in the present study (433 mg/kg DMI) compared with the value derived from gelatin-based P-free diet in the previous study (560 mg/kg DMI) [37] was partly attributed to different dietary Ca concentrations. A decreased serum Ca level leads to the release of parathyroid hormone that stimulates bone resorption and increases urinary P loss [32]. Considering the greater dietary Ca concentration in the P-free diet in the current study (0.88%) compared with the gelatin-based diet (0.08%), the influence of dietary Ca on the BEL of P at the total tract would have been less in the present study, resulting in a lower BEL of P at the total tract.

## 5. Conclusions

The ileal digestibility of P determined by the direct method was greater than total tract retention in inorganic phosphates fed to broiler chickens. As the P utilization of inorganic phosphates varies in chickens, different P utilization values should be used for each inorganic phosphate when formulating poultry diets.

## Figures and Tables

**Table 1:** Analyzed calcium and phosphorus concentrations in inorganic phosphates (as-is basis).

Item %	MCP	MDCP	DCP	TCP
Calcium	16.2	16.3	23.5	38.4
Phosphorus	22.3	21.4	18.5	20.1

MCP = monocalcium phosphate; MDCP = monodicalcium phosphate; DCP = dicalcium phosphate; and TCP = tricalcium phosphate.

**Table 2:** Ingredients and chemical compositions of the experimental diets (as-fed basis).

Item	Experimental Diet				
	MCP	MDCP	DCP	TCP	P-Free
Ingredient, %					
MCP	2.06	0.00	0.00	0.00	0.00
MDCP	0.00	2.49	0.00	0.00	0.00
DCP	0.00	0.00	2.44	0.00	0.00
TCP	0.00	0.00	0.00	2.50	0.00
Limestone	1.55	1.42	1.00	0.43	2.41
Gelatin	17.50	17.50	17.50	17.50	17.50
Cornstarch	42.73	42.43	42.90	43.41	43.93
Sucrose	20.00	20.00	20.00	20.00	20.00
Cellulose	5.00	5.00	5.00	5.00	5.00
Soybean oil	2.00	2.00	2.00	2.00	2.00
Amino acid mixture ^1^	5.81	5.81	5.81	5.81	5.81
Sodium bicarbonate	0.80	0.80	0.80	0.80	0.80
Potassium chloride	0.30	0.30	0.30	0.30	0.30
Potassium carbonate	0.29	0.29	0.29	0.29	0.29
Choline chloride	0.26	0.26	0.26	0.26	0.26
Sodium chloride	0.10	0.10	0.10	0.10	0.10
Magnesium oxide	0.10	0.10	0.10	0.10	0.10
Vitamin premix ^2^	0.50	0.50	0.50	0.50	0.50
Mineral premix ^3^	0.50	0.50	0.50	0.50	0.50
Chromic oxide	0.50	0.50	0.50	0.50	0.50
Calculated composition, %					
Crude protein	20.0	20.0	20.0	20.0	20.0
Lysine	1.42	1.42	1.42	1.42	1.42
Methionine and cysteine	0.99	0.99	0.99	0.99	0.99
Calcium	1.00	1.00	1.00	1.00	1.00
Phosphorus	0.45	0.45	0.45	0.45	0.00
Sodium	0.26	0.26	0.26	0.26	0.26
Potassium	0.22	0.22	0.22	0.22	0.22
Analyzed composition					
Dry matter, %	94.3	94.2	94.4	94.8	94.7
Gross energy, kcal/kg	4039	3985	3995	4011	4026
Calcium, %	1.05	1.08	1.03	1.00	0.89
Phosphorus, %	0.44	0.50	0.43	0.49	0.01

MCP = monocalcium phosphate; MDCP = monodicalcium phosphate; DCP = dicalcium phosphate; and TCP = tricalcium phosphate. ^1^_L_-arginine, 0.08%; _L_-histidine, 0.35%; _L_-isoleucine, 0.65%; _L_-leucine, 0.88%; _L_-lysine, 0.82%; _DL_-methionine, 0.39%; _L_-cysteine, 0.47%; _L_-phenylalanine, 0.62%; _L_-threonine, 0.66%; _L_-tryptophan, 0.20%; and _L_-valine, 0.69%. ^2^ The following quantities per kilogram of complete diet were provided: vitamin A, 60,000 IU; vitamin D_3_, 20,000 IU; vitamin E, 400 IU; vitamin K_3_, 20 mg; vitamin B_1_, 20 mg; vitamin B_2_, 50 mg; vitamin B_3_, 300 mg; vitamin B_6_, 30 mg; vitamin B_12_, 0.1 mg; biotin, 1.0 mg; pantothenic acid, 100 mg; and folic acid, 10 mg. ^3^ The following quantities per kilogram of complete diet were provided: copper, 80 mg as copper sulfate; iron, 300 mg as iron sulfate; iodine, 6 mg as calcium iodate; manganese, 600 mg as manganese sulfate; zinc, 500 mg as zinc sulfate; cobalt, 5 mg as cobaltous carbonate; and selenium, 2 mg as sodium selenite.

**Table 3:** Growth performance of broiler chickens fed experimental diets ^1^.

Item	Experimental Diet				SEM	*p*-Value
	MCP	MDCP	DCP	TCP		
Initial BW, g	399	399	399	399	14	0.177
Final BW, g	411 ^a^	403 ^ab^	397 ^b^	397 ^b^	13	0.002
ADG, g/d	3.1 ^a^	0.9 ^ab^	−0.5 ^b^	−0.6 ^b^	0.8	0.001
ADFI, g/d	20.4 ^a^	20.0 ^ab^	18.0 ^b^	19.2 ^ab^	0.6	0.041
Gain to feed ratio	0.147 ^a^	0.045 ^ab^	−0.029 ^b^	−0.032 ^b^	0.040	0.002

MCP = monocalcium phosphate; MDCP = monodicalcium phosphate; DCP = dicalcium phosphate; TCP = tricalcium phosphate; SEM = standard error of the means; BW = body weight; ADG = average daily gain; and ADFI = average daily feed intake. ^a,b^ Least squares means within a row without a common superscript differ (*p* < 0.05). ^1^ Each least squares mean represents eight observations.

**Table 4:** Apparent ileal digestibility (AID) and apparent total tract retention (ATTR) of phosphorus (P) in inorganic phosphates fed to broiler chickens.

Item, %	MCP	MDCP	DCP	TCP	RMSE	*p*-Value ^2^		
						P Source	Collection Site	Interaction
AID of P ^1^	86.7 ^a^	86.0 ^a^	76.2 ^ab^	53.8 ^c^	9.33	<0.001	<0.001	0.652
ATTR of P ^1^	64.0 ^ab^	60.0 ^c^	57.4 ^c^	30.7 ^d^				
Mean	75.4 ^a^ (16)	73.0 ^ab^ (16)	66.8 ^b^ (16)	42.3 ^c^ (8)				

MCP = monocalcium phosphate; MDCP = monodicalcium phosphate; DCP = dicalcium phosphate; TCP = tricalcium phosphate; and RMSE = root mean square error. ^a–d^ Least squares means within a row without a common superscript differ (*p* < 0.05). ^1^ Each least squares mean represents eight observations except for TCP (four observations). ^2^ Collection site = comparison between ileal digestibility and total tract retention; Interaction = interaction between the P source and the collection site.

**Table 5:** Standardized ileal digestibility (SID) and standardized total tract retention (STTR) of phosphorus (P) in inorganic phosphates fed to broiler chickens ^1^.

Item, %	MCP	MDCP	DCP	TCP	RMSE	*p*-Value ^3^		
						P Source	Collection Site	Interaction
SID of P ^2^	89.8 ^a^	88.7 ^a^	79.5 ^ab^	56.7 ^cd^	9.33	<0.001	<0.001	0.552
STTR of P ^2^	73.3 ^b^	68.2 ^bc^	67.0 ^bc^	39.1 ^d^				
Mean	81.6 ^a^ (16)	78.5 ^ab^ (16)	73.2 ^b^ (16)	47.9 ^c^ (8)				

MCP = monocalcium phosphate; MDCP = monodicalcium phosphate; DCP = dicalcium phosphate; TCP = tricalcium phosphate; and RMSE = root mean square error. ^a–d^ Least squares means within a row without a common superscript differ (*p* < 0.05). ^1^ SID and STTR values were calculated by correcting the AID and ATTR values for the basal endogenous loss of P at the ileum and total tract, respectively. The basal endogenous loss of P at the ileum [145 ± 47 mg/kg of dry matter (DM) intake] was less (*p* < 0.05) than that at the total tract (433 ± 141 mg/kg of DM intake). ^2^ Each least squares mean represents eight observations except for TCP (four observations). ^3^ Collection site = comparison between ileal digestibility and total tract retention; Interaction = interaction between the P source and the collection site.
